# Statins use and the risk of all and subtype hematological malignancies: a meta-analysis of observational studies

**DOI:** 10.1002/cam4.411

**Published:** 2015-03-21

**Authors:** Danitza Pradelli, Davide Soranna, Antonella Zambon, Alberico Catapano, Giuseppe Mancia, Carlo La Vecchia, Giovanni Corrao

**Affiliations:** 1 Department of Statistics and Quantitative Methods, Division of Biostatistics, Epidemiology and Public Health, University of Milano-BicoccaMilan, Italy; 2 Department of Pharmacological Sciences, University of MilanoMilan, Italy; 3 IRCCS, Multimedica, MilanItaly; 4 Department of Clinical Medicine and Prevention, University of Milano-Bicocca, Monza (MB)Italy; 5 Department of Epidemiology, Institute for Pharmacological Research Mario NegriMilan, Italy; 6 Department of Clinical Sciences and Public Health, University of MilanoMilan, Italy

**Keywords:** Hematologic malignancies, leukemia, myeloma, non-Hodgkin lymphoma, statins

## Abstract

In order to quantify the association between use of statins and the risk of all hematological malignancies and of subtypes, we performed a meta-analysis of observational studies. We achieved a MEDLINE/EMBASE comprehensive search for studies published up to August 2014 investigating the association between use of statins and the risk of hematological malignancies, including Hodgkin- and non-Hodgkin lymphoma, leukemia, and myeloma. Fixed- and random-effect models were fitted to estimate the summary relative risk (RR) based on adjusted study-specific results. Between-study heterogeneity was assessed using the Q and *I*^2^ statistics and the sources of heterogeneity were investigated using Deeks' test. Moreover, an influence analysis was performed. Finally, publication bias was evaluated using funnel plot and Egger's regression asymmetry test. Fourteen studies (10 case–control and four cohort studies) contributed to the analysis. Statin use, compared to nonuse of statins, was negatively associated with all hematological malignancies taken together (summary RR 0.86; 95% CI: 0.77–0.96), with leukemia (0.83; 0.74–0.92), and non-Hodgkin lymphoma (0.81; 0.68 to 0.96), but it was not related to the risk of myeloma (0.89; 0.53–1.51). Long-term users of statins showed a statistically significant reduction in the risk of all hematological malignancies taken together (0.78; 0.71–0.87). Statistically significant between-studies heterogeneity was observed for all outcome except for leukemia. Heterogeneity was caused by differences confounding-adjustment level of the included studies only for Myeloma. No significant evidence of publication bias was found.

## Introduction

Statins (HMG-CoA-reductase inhibitors) are the most commonly prescribed drugs worldwide to reduce plasma cholesterol levels due to their cardiovascular protective effects and excellent tolerability 1–4 and their use has increased strikingly in the past decade 5. Recent in vivo investigations have suggested that these drugs may have a chemopreventive potential against hematopoietic and lymphatic malignancies 6–8. A study on humans showed a protective effect on non-Hodgkin lymphoma in subjects affected by the genetic deficiency of glucose-6-phosphate dehydrogenase leading to the reduced availability of the NADPH, required for the activity of 3-hydroxy-3-methylglutaryl CoA reductase 9. Some observational studies reported decreased non-Hodgkin lymphoma risk of 26–45% in users of statins 10,11. A protective effect on the risk of hematological malignancies of the same strength (24%) was reported for long-term use of statins versus short-term use of statins 12. Moreover, a reduction in the multiple myeloma risk of 60% 13 and in the leukemia risk of 26% 14 for any use of statins was showed. However, inconsistent findings were retrieved from meta-analytic approach. A meta-analysis, based on six randomized trials and eight observational studies, did not support a potential role of statins in the prevention of any hematological malignancies 15 while a recent meta-analysis, based on 14 observational studies, showed chemopreventive effects against hematological malignancies 16. Moreover, to our knowledge only a relatively dated meta-analysis had evaluated the effect of statins on the risk of specific hematological cancer. This meta-analysis considered a few studies for specific hematological malignancies and showed a protective effect only for lymphoma (median relative risk [RR] 0.74, range 0.28–2.2) 17.

Thus, the effect of statins on the risk of all and subtype hematological malignancies remains to be determined. To address this issue, we carried out a meta-analysis of available observational studies published on this topic.

## Methods

### Search strategy and study selection

We carried out a MEDLINE and EMBASE search for observational studies published up to August 2014 which investigated the association between “statin” and risk of “hematological malignancies.”

The following keywords and/or corresponding MeSH terms were used: (“Hydroxymethylglutaryl-CoA reductase inhibitors” OR “HMG-CoA-reductase inhibitors” OR “statin” OR “simvastatin” OR “pitavastatin” OR “lovastatin” OR “fluvastatin” OR “pravastatin” OR “atorvastatin” OR “rosuvastatin”) AND (“hematologic malignancies” OR “hematologic neoplasms” OR “hematopoietic malignancies” OR “hematopoietic neoplasms” OR “lymphoma” OR “leukemia” OR “myeloma”). In addition, the reference lists of reviews and meta-analyses published on this issue, identified in MEDLINE and Cochrane Library, were hand-checked to find additional relevant publications 15–23.

All identified titles and abstracts were accurately scanned to exclude studies that did not fit inclusion criteria. Cohort and case–control studies were both included, provided that they (1) investigated any use of statin and that explicitly considered nonusers of statins as the reference category; (2) considered as outcome of the following events: hematological malignancy as a whole and/or specific malignancies such as Hodgkin- and non-Hodgkin lymphoma, leukemia, and myeloma; (3) reported crude or adjusted estimates of the association between exposure and outcome (odds ratio [OR], or hazard ratio [HR] considered as RRs 24 and their corresponding 95% CI or *P*-value) or sufficient data to calculate them.

When data were published more than once, the most recent and complete publication was considered. Two readers (D. P. and D. S.), independently determined the eligibility of each article for inclusion. Discrepancies between readers were resolved in conference.

### Data collection

The following data were collected from each included article: publication year, study design, country, source of data, characteristics of the subjects (e.g., gender), number of cases, cancer type, control for confounding factors (matching or adjustments), and estimates for exposure–outcome relationship together with corresponding 95% confidence interval (CI) or *P*-value.

### Statistical analysis

The summary RR for use of statin versus no use (including never use and short duration of statin use) and risk of all and subtype hematological malignancies was the main measure of interest. Analyses were performed for hematological malignancies as a whole, as well as for each subtype, provided that the corresponding estimates were available in at least three studies. Where possible, we included in the analysis the adjusted estimates of the RR from the original studies; otherwise we used raw data and computed unadjusted RRs.

The dose–response analysis was performed only for articles where the association estimates for “long-term users” considered a treatment period longer than 4 years versus no users.

Between-study heterogeneity was tested by Cochran's Q test 25 and measured with the *I*^2^ statistics (the proportion of between-study variability caused by heterogeneity) 26. We pooled the original estimates by using both the Mantel & Haenszel method (fixed-effects model) and the DerSimonian & Laird method (random-effects model) 27. When a significant heterogeneity was found, the results from the random-effects model were showed. Between-study sources of heterogeneity were investigated by stratifying original estimates according to some study characteristics potentially relevant in causing heterogeneity, that is, study design (cohort or case–control), geographic area (Europe, Other countries), level of control for possible confounders (low: only sociodemographic characteristics; high: sociodemographic and other variables or no adjusted). The Deeks test was used to evaluate the significancy of the difference between subgroups 27. An influence analysis was also conducted by omitting one study at a time, in order to identify to what extent the results were influenced by a single study. Publication bias was evaluated through funnel plot visual analysis and the Egger's test 28.

All tests were considered statistically significant for *P*-values less than 0.05. The analyses and the corresponding graphical visualization of forest and funnel plots were, respectively, conducting using Review Manager (RevMan 5.1) (Nordic Cochrane Center) and STATA Software Program Version 9 (STATA, College Station, TX). The PRISMA statements were taken into account in this paper 29.

## Results

**Figure 1:**
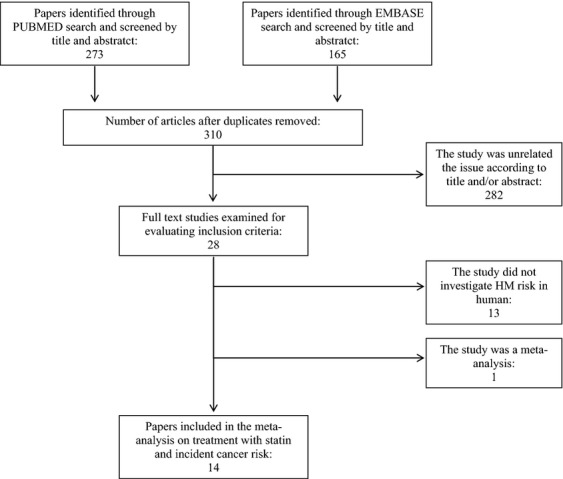
Flowchart of the selection of studies for inclusion in the meta-analysis.

Figure1 shows the flow diagram for study inclusion. Based on title and abstract the PUBMED search allowed to identify 273 papers, further 165 papers were retrieved by EMBASE search. After the duplicate removal, we considered 310 studies. We excluded 282 papers because they were unrelated to the issue and further 14 papers because they did not satisfy the inclusion criteria. The remaining 14 studies 10–14,30–38 were considered for meta-analysis. Table1 shows that these comprised four cohort and 10 case–control studies on a total of 17,886 patients with hematological malignancies (irrespectively from their subtype), of which 1174 with leukemia (five studies; for one study 14 the number of cases was not available), 3469 with non-Hodgkin lymphoma (seven studies), and 609 myelomas (four studies).

**Table 1:** Chronological summary of literature on use of statins and risk of hematologic malignancies, and their main characteristics

First author, country [reference]	Study design	Source of data	Gender	Hematologic malignancy subtype	No. of cases	Reported RR (95% CI)	Controlled variables/notes
Friis et al., Denmark 30	Cohort	Population	MW	Lymphatic/hematopoietic	1626	0.88 (0.60–1.29)	Age, sex, calendar period, use of NSAID, use of HRT, use of cardiovascular drugs
Friedman et al., USA 31	Cohort	Population	M	HL	13	1.19 (0.66–2.13)	Smoking, use of NSAIDs, calendar year
				Lymphocytic leukemia	42	0.96 (0.69–1.33)	
				Lymphocytic leukemia	19	0.82 (0.51–1.32)	
				Mieloid leukemia	44	0.86 (0.63–1.19)	
				Mieloid leukemia	26	1.02 (0.68 to 1.54)	
				Multiple myeloma	49	0.83 (0.61–1.12)	
				Multiple myeloma	41	1.03 (0.74 to 1.43)	
				NHL	164	0.94 (0.80–1.11)	
				NHL	118	0.95 (0.78–1.15)	
Jacobs et al., USA 10	Cohort	Population	MW	NHL	59	0.84 (0.72–0.98)	Age, sex, ethnicity, education, smoking, use of NSAIDs, BMI, physical activity level, nonsteroidal anti-inflammatory drug use, hormone therapy, history of elevated cholesterol, heart disease, diabetes, hypertension
Lutski et al., Israel 12	Cohort	Population	MW	Hematological malignancies	681	0.69 (0.55–0.88)	Age, sex, marital status, area of residence, nationality, socioeconomic level, years of stay in Israel, obesity, diabetes mellitus, hypertension, cardiovascular disease, efficacy, hospitalizations, and visits to physicians a year before first statin dispensation and asthma
				Leukemia	177	0.58 (0.37–0.91)	
				Lymphoma	429	0.69 (0.51 to 0.94)	
Traversa et al., Italy 32	Case–control	Population	MW	Acute leukemia	202	1.50 (0.80 to 2.60)	Age, sex
Blais, Canada 33	Case–control	Population	MW	Lymphoma	24	2.17 (0.38–12.36)	Age, sex, use of fibric acid, previous benign neoplasm, year of cohort entry, the score of comorbidity
Graaf et al., Netherland 34	Case–control	Population	MW	Lymphoma	93	0.28 (0.06–1.30)	Age, sex, geographic region, follow-up time, calendar time, diabetes mellitus, prior hospitalizations, chronic disease score, chronic use of diuretics; ACE-I, calcium channel blockers, hormones, NSAIDs, other LLT, familiar hypercholesterolemia
Zhang et al., USA 35	Case–control	Population	W	NHL	601	0.50 (0.40–0.80)	Age, BMI, menopausal status, and family history of NHL in first degree relatives
Fortuny et al., Europe 36	Case–control	Hospital cases and hospital or population controls	MW	Lymphoma	2,362	0.61 (0.45–0.84)	Age, gender, country
				NHL (B-cell L)	1,858	0.61 (0.44–0.84)	
				NHL (T cell L)	136	0.74 (0.29–1.86)	
				Leukemia (CLL-SLL)	410	0.83 (0.51–1.34)	
				Myeloma	281	0.47 (0.22–0.99)	
Iwata et al., Japan 37	Case–control	Hospital	MW	Lymphoid malignancies	221	2.24 (1.37–3.66)	Age, sex, year of visit, serological status for anti-Hepatitis B surface antigens (HBsAg) and anti-Hepatitis C virus antibodies (HCVAb)
				DLBL (NHL)	66	2.10 (0.79–5.55)	
				FL (NHL)	28	1.94 (0.35–10.90)	
				Plasma cell. myeloma	59	3.99 (1.75–9.10)	
Landgren et al., USA 13	Case–Control	Population	W	Myeloma	179	0.40 (0.20–0.80)	Age, race, education, BMI
Coogan et al., USA 38	Case–Control	Hospital	MW	Leukemia	254	1.10 (0.60–2.00)	Age, sex, BMI, interview year, study center, alcohol use, race, years of educational, pack-years of smoking. NSAID use
				NHL	144	1.20 (0.60–2.40)	
Chao et al., USA 11	Case–Control	HIV population	MW	NHL	295	0.55 (0.31–0.95)	Age, sex, ethnicity, index year, know duration of HIV infection, Kaiser Permanente Region, clinical Aids priori to index date, duration of antiretroviral therapy use, baseline CD4 cell count level, history of hepatitis B and C, diabetes, and obesity
Vinogradova et al., UK 14	Case–Control	Population	MW	Hematological malignancies	7185	0.78 (0.71–0.86)	Townsend quintile, BMI, smoking, myocardial infarction, coronary heart disease, diabetes, hypertension, stroke, rheumatoid arthritis, use of NSAIDs, Cox2-inhibitors, aspirin
				Leukemia		0.74 (0.62–0.87)	

LLT, lipid-lowering therapy; HRT, hormone replacement therapy; NHL, non-Hodgkin lymphoma; CLL, chronic lymphocytic leukemia; SLL, small cell lymphocytic leukemia; DLBL, diffuse large B-cell lymphoma; FL, follicular lymphoma; HL, Hodgkin lymphoma; L, lymphoma; M, men; W, women.

**Figure 2:**
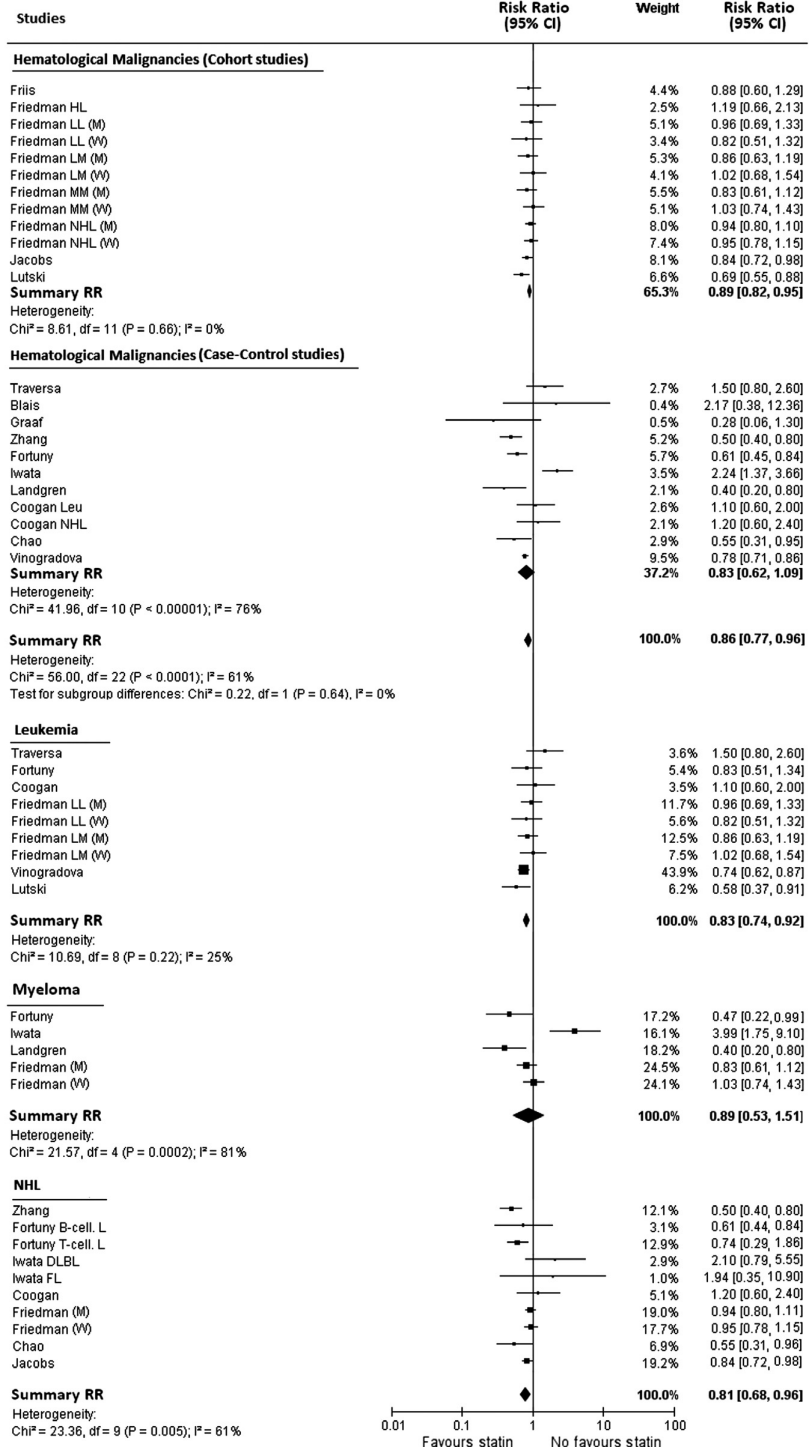
Study-specific and summary relative risk estimates for the association between use of statins and the risk of all hematological malignancies taken together, leukemia, myeloma, and non-Hodgkin lymphoma. Squares represent study-specific relative risk estimates (size of the square reflects the study-specific statistical weight, i.e., the inverse of the variance); horizontal lines represent 95% CIs; diamonds represent summary relative risk estimates with corresponding 95% CIs; *P*-values are from testing for heterogeneity across study-specific estimates.

Figure2 shows the study-specific and summary RR for use versus nonuse of statins. The summary RR for all hematological malignancies irrespectively from their subtype was 0.86 (95% CI: 0.77–0.96) without statistically significant difference (Deeks test *P*-value 0.64) between cohort (summary RR, 0.89; 95% CI: 0.82–0.95) and case–control (summary RR, 0.83; 95% CI: 0.62–1.09) studies.

There was no statistically significant association (the corresponding summary RR, and 95% CI, being 0.89, 0.53–1.51) for myeloma, but there was a significant between-study heterogeneity (Chi^2^ test *P*-value and *I*^2^ statistics being 0.0002 and 81%).

A statistically significant reduction in the risk was observed for both, leukemia (summary RR 0.83; 95% CI: 0.74–0.92), and non-Hodgkin lymphoma (summary RR 0.81; 95% CI: 0.68–0.96) with a statistically significant between-study heterogeneity only for the latter one (Chi^2^ test *P*-value and *I*^2^ statistics, respectively, of 0.005 and 61% for non-Hodgkin lymphoma and of 0.220 and 25% for leukemia).

In the stratified analysis performed to identify the sources of heterogeneity, only the different level of control for possible confounders showed evidence of modifying the summary analysis of Myeloma (*P*-value 0.0002).

These results were partially influenced by omitting one study at a time. A statistically nonsignificant reduction in the risk of non-Hodgkin lymphoma was observed in statin users omitting the studies of Fortuny et al. 36 or of Jacobs et al. 10 with new summary RRs, respectively, of 0.84 (95% CI: 0.70–1.02) and 0.80 (95% CI: 0.64–1.01). Analogously, for leukemia, the exclusion of Vinogradova et al. study 14 nullified the association with a new summary RR of 0.90 (95% CI: 0.77 to 1.05). Finally, for myeloma the omission of the study of Fortuny et al. 36 or of Landgren et al. 13 nullified the potential protective effect of statins with new summary RRs estimates, respectively, of 1.02 (95% CI: 0.57–1.83) and 1.06 (95% CI: 0.61–1.84).

Since influence effects were observed for both hospital (Fortuny et al. 36) and/or population-based design (Jacobs et al. 10, Vinogradova et al. 14, Landgren et al. 13) we think that our estimates are light affected by source of data of included studies. Moreover, the Iwata study showed the more elevated risk but its influence was limited (weight 4-16%).

**Figure 3:**
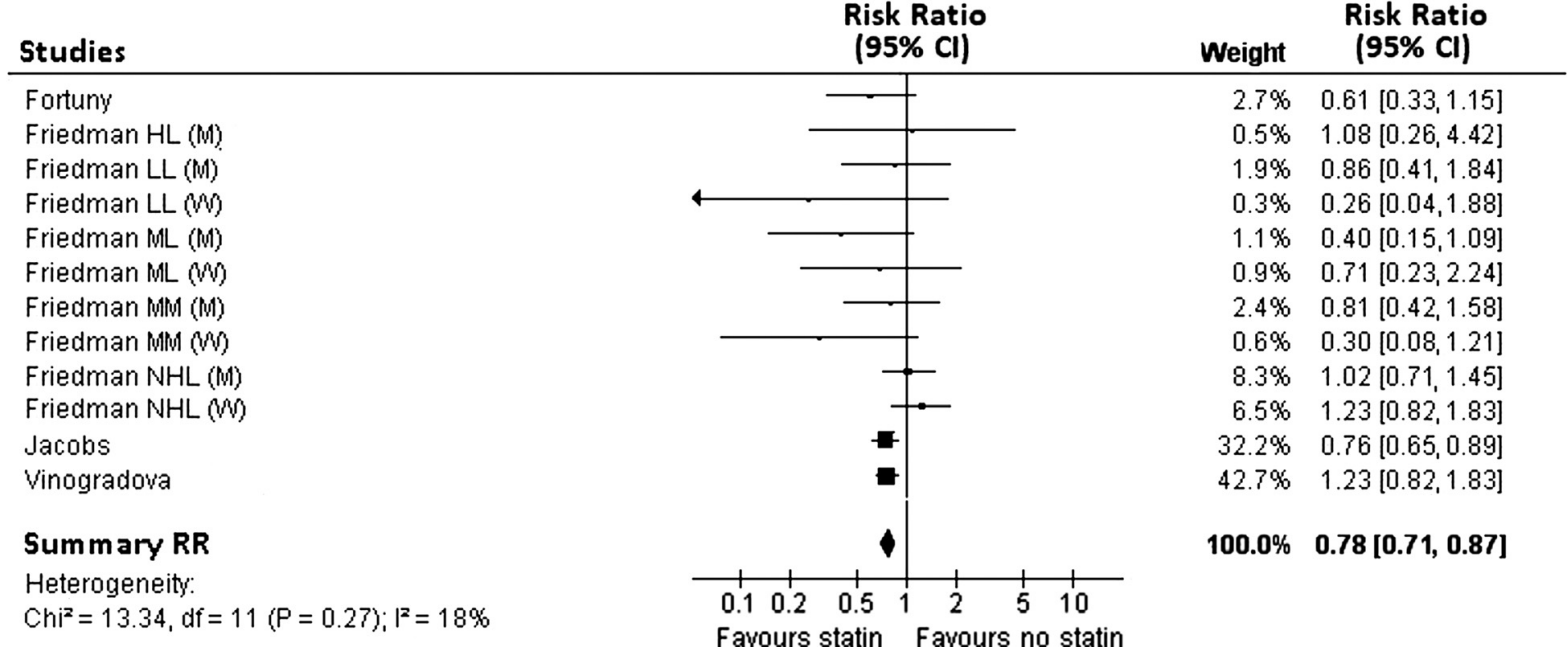
Study-specific and summary relative risk estimates for the association between “long-term” use of statins and the risk of hematological malignancies. Squares represent study-specific relative risk estimates (size of the square reflects the study-specific statistical weight, i.e., the inverse of the variance); horizontal lines represent 95% CIs; diamonds represent summary relative risk estimates with corresponding 95% CIs; *P*-values are from testing for heterogeneity across study-specific estimates.

Figure3 shows the study-specific and summary RR of all hematological malignancies associated with “long-term use” of statins. A statistically significant reduction in the risk was observed with a summary RR of 0.78, 95% CI: 0.71–0.87, without any evidence of between-study heterogeneity (Chi^2^ test *P*-value 0.270 and *I*^2^ = 18%).

**Figure 4:**
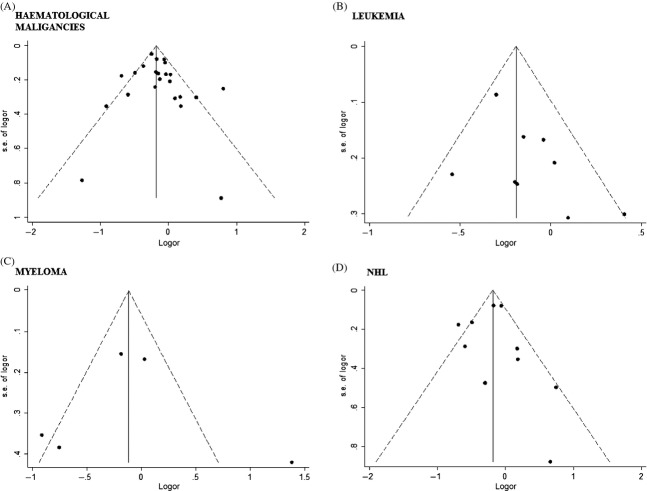
Funnel plot for publication bias of studies investigating the association between use of statins and the risk of all hematological malignancies taken together (A), leukemia (B), myeloma (C), and non-Hodgkin lymphoma (D).

There was some evidence of publication bias from visualization of the funnel plot (Fig.4), but this was not confirmed from corresponding Egger's test (hematological malignancies *P*-value = 0.453, leukemia *P*-value = 0.120, myeloma *P*-value = 0.983, and non-Hodgkin lymphoma *P*-value = 0.904.

## Discussion

We analyzed the data from 14 observational studies in order to evaluate the effect of statins on the risk of both all and subtype hematological malignancies. Our comprehensive meta-analysis showed a statistically significant reduction in the risk of hematological malignancies according to the meta-analytic results of Yi et al. 16. Moreover, in our study a statistically significant risk reduction from summarizing estimates associated with “long-term use” of statins was observed scoring in favor of the hypothesis of a causal association between chronic use of statins and hematological malignancies.

Two relevant studies: (1) a study of six randomized clinical trials eight observational studies; (2) a pooled individual-level data of 27 randomized trials that did not show any effect of statin therapy on the risk of all hematological malignancies 16,39. The inconsistency of this findings with our results could be caused by the small number of observational studies included (only eight studies) and by the fact that randomized controlled trials may not be appropriate for the assessment of rare outcomes or effects that take a long time to develop, in fact total number of hematological malignancies in all 27 eligible RCTs was 614, compared to 17,866 in the current meta-analysis of observational studies 40.

Analyzing specific subtype hematological malignancies we observed a potential protective effect for leukemia (summary RR 0.83; 95% CI: 0.74–0.92) and non-Hodgkin lymphoma (summary RR 0.81; 95% CI: 0.68–0.96), although the high between-studies heterogeneity for the latter outcome suggests that the findings should still be regarded as inconclusive.

Our results are consistent with several previous findings. Two in vitro studies showed that statins suppress the growth of promyelocytic and lymphocytic leukemic cells 41,42. One study conducted on 28 inbred rats showed that treatment with Lovastatin caused inhibition of spontaneous metastasis of poorly differentiated lymphomas without affecting primary tumor growth 6. Experimental cancer models have shown that Lovastatin induces a profound apoptotic response in cells derived from juvenile monomyelocytic leukemia. Tumor cells themselves differ significantly in their sensitivity to statin-induced cell death: myeloblastic leukemia cells and neuroblastoma cells seem to be particularly sensitive to statin-induced apoptosis, whereas acute lymphoblastic leukemia cells are relatively insensitive 43.

The strength of the evidence for the effect of statins use on leukemia is reduced by the observation that the result was modified by the omission of the most relevant study on this issue (summary RR 0.90 [95% CI: 0.77–1.05]) 14. However, if the selective inclusion with protective effect of statins on the risk of leukemia, suggested by funnel plot, were real our association measurements could be underestimated. Moreover, selective exclusion of the so-called “grey literature” (PhD theses, abstracts, conference proceedings, etc.) might also play a role. Nevertheless, the results that statins may exert a protective effect on the risk of leukemia call for a greater attention to this important issue in future studies.

Finally, no evidence of protective effect of statins use on myeloma was reported (summary RR 0.89, 95% CI: 0.53–1.51) perhaps due to the small number of studies and the high between-studies heterogeneity.

Our results have limitations which mainly reflect the sources of bias of the observational studies included into the meta-analysis. In particular, observational investigations lacked random allocation of the intervention necessary to correctly investigate exposure–outcome causal relationship. As a result, we cannot exclude the possibility that confounding by indication might explain our findings. Despite primary studies reported estimates adjusted for the history of several medical conditions associated with statin use that might also affect hematological malignancies risk, residual confounding remains a potential limitation.

Furthermore, since little is known about the etiology of hematological malignancies, we cannot rule out unknown confounders as possible explanation for our findings. The definition of outcome varied from study to study. Combining studies would increase the power for a given hematological malignancy subtype, but the heterogeneity would also have increased. Further, cholesterol levels influence statin use as well as possibly modifying cancer risk, though data are inconsistent 44. The decreased risk of hematological malignancies could be explained by reverse causality, as patients with such diagnoses are more likely to have lower lipid levels 45. Another limitation was the inability to evaluate the effect of various types of statins, given the considerable variation in their bioavailability 46.

## Conclusion

Given the widespread and rapidly increasing use of statins, any association with an increased or decreased risk of no cardiovascular disease would have substantial public health impact. Our study provides evidence that statins seem to reduce the risk of hematological malignancy. We also found that statins users had a statistically significant reduced risk of leukemia and non-Hodgkin lymphoma than nonusers. Moreover, evidence on long-term effects of statins on hematological malignancies is available. These evidences, although not conclusive because based on a small number of studies included in this meta-analysis and characterized by a strong heterogeneity among study-specific association estimates are interesting signals on a secondary potential benefit of statins therapy.
